# To Make or Take: Bacterial Lipid Homeostasis during Infection

**DOI:** 10.1128/mBio.00928-21

**Published:** 2021-06-17

**Authors:** Felise G. Adams, Claudia Trappetti, Jack K. Waters, Maoge Zang, Erin B. Brazel, James C. Paton, Marten F. Snel, Bart A. Eijkelkamp

**Affiliations:** a Molecular Sciences and Technology, College of Science and Engineering, Flinders University, Adelaide, South Australia, Australia; b Research Centre for Infectious Diseases, School of Biological Sciences, University of Adelaide, Adelaide, South Australia, Australia; c Proteomics, Metabolomics and MS-Imaging Core Facility, South Australian Health and Medical Research Institute, Adelaide, South Australia, Australia; Louis Stokes Veterans Affairs Medical Center

**Keywords:** *Acinetobacter*, lipids, fatty acids, membrane biogenesis, pathogenesis

## Abstract

Bacterial fatty acids are critical components of the cellular membrane. A shift in environmental conditions or in the bacterium’s lifestyle may result in the requirement for a distinct pool of fatty acids with unique biophysical properties. This can be achieved by the modification of existing fatty acids or via *de novo* synthesis. Furthermore, bacteria have evolved efficient means to acquire these energy-rich molecules from their environment. However, the balance between *de novo* fatty acid synthesis and exogenous acquisition during pathogenesis is poorly understood. Here, we studied the mouse fatty acid landscape prior to and after infection with Acinetobacter baumannii, a Gram-negative, opportunistic human pathogen. The lipid fluxes observed following infection revealed fatty acid- and niche-specific changes. Lipidomic profiling of A. baumannii isolated from the pleural cavity of mice identified novel A. baumannii membrane phospholipid species and an overall increased abundance of unsaturated fatty acid species. Importantly, we found that A. baumannii relies largely upon fatty acid acquisition in all but one of the studied niches, the blood, where the pathogen biosynthesizes its own fatty acids. This work is the first to reveal the significance of balancing the making and taking of fatty acids in a Gram-negative bacterium during infection, which provides new insights into the validity of targeting fatty acid synthesis as a treatment strategy.

## OBSERVATION

The bacterial type II fatty acid synthesis (FASII) pathway has been interrogated as a potential antimicrobial target, with the validity of this strategy being debated through studies of the Gram-positive bacterium Staphylococcus aureus (1, 2). In addition to FASII, pathogens can readily acquire fatty acids from their environment through either the FakAB system (Gram-positive bacteria) (3) or FadL (Gram-negative bacteria) (4, 5), which may render them resistant to FASII-targeting antimicrobial strategies, as illustrated in S. aureus (6, 7). Nevertheless, S. aureus fatty acid auxotrophs are avirulent in a mouse model of disease (8), indicating that at least some level of *de novo* synthesis is critical for bacterial survival during infection. Interestingly, the balance between *de novo* fatty acid synthesis and exogenous acquisition has never before been studied in Gram-negative bacterial pathogens. Furthermore, the niche specificity of bacterial lipid homeostasis is poorly understood.

**FIG 1:**
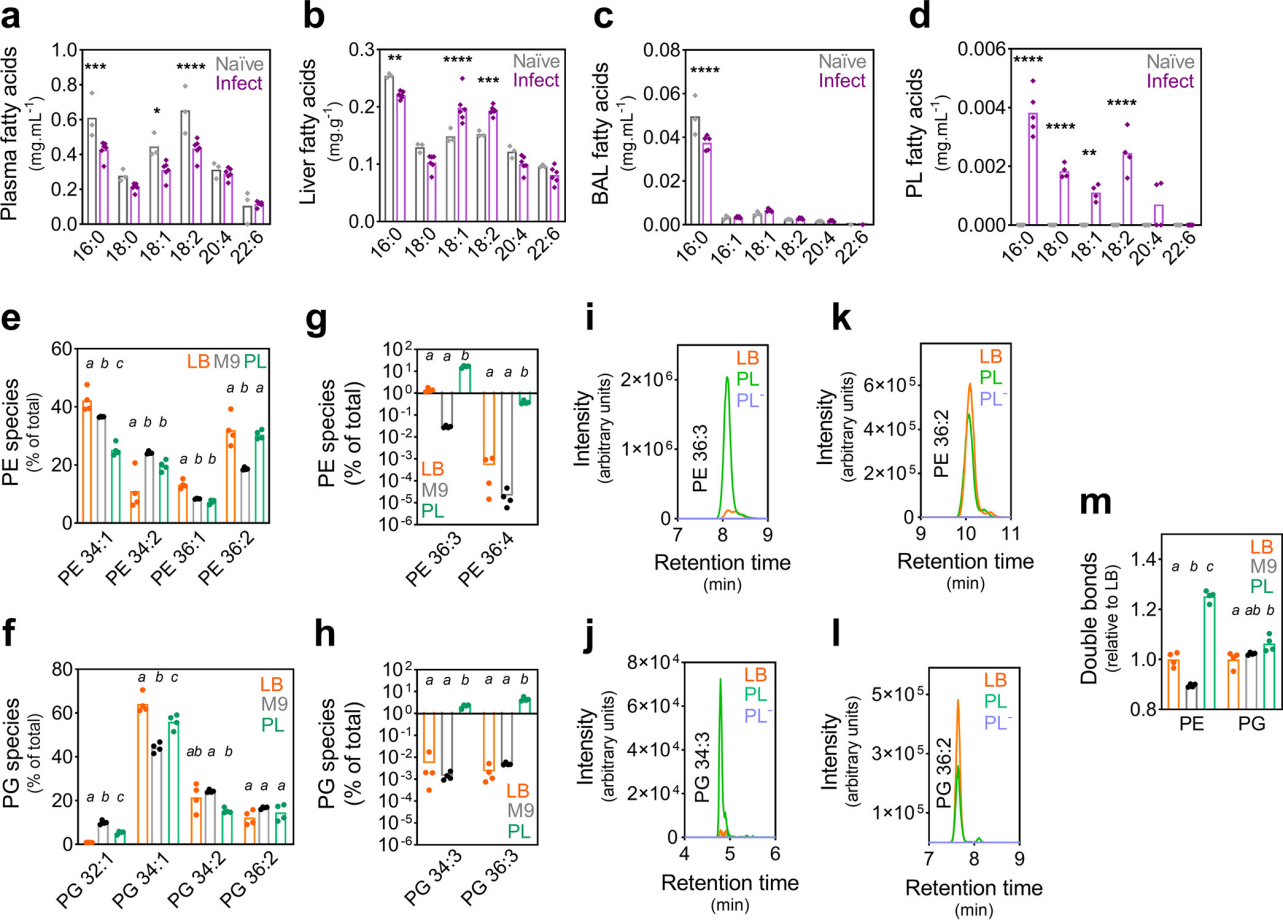
The host and A. baumannii lipid landscape. (a to d) The fatty acids (milligrams of fatty acid per milliliter or gram of tissue) in the plasma (a), liver tissue (b), bronchoalveolar lavage (BAL) fluid (c), and pleural lavage (PL) fluid (d) of 9-week-old female BALB/c mice were examined prior to (Naïve) and 24 h after (Infect) intranasal challenge with A. baumannii strain AB5075_UW. Statistical analyses were performed using one-way analysis of variance (ANOVA) (Bonferroni test). *, *P* ≤ 0.05; **, *P* ≤ 0.01; ***, *P* ≤ 0.001; ****, *P* ≤ 0.0001. (e to h) The phosphatidylethanolamine (PE) (e and g) and phosphatidylglycerol (PG) (f and h) species (number of carbons:number of double bonds in the acyl chains) were quantified in A. baumannii cultured in Luria-Bertani (LB) or M9 medium or A. baumannii from the pleural lavage fluid of BALB/c mice 24 h after intranasal challenge, using liquid chromatography-mass spectrometry (LC-MS). (i to l) LC-MS chromatograms for heavily enriched lipid species in A. baumannii isolated from the PL fluid (PE 36:3 [i] and PG 34:3 [j]) and control lipid species present in A. baumannii from the PL fluid and those cultured in LB medium (PE 36:2 [k] and PG 36:2 [l]). The PL fluid from uninfected mice (PL^−^) is included as a control (no discernible signal). The data represent the averages from 4 biological replicates. (m) The relative number of double bonds of PE and PG species in A. baumannii from LB or M9 media, or the PL fluid was defined using the percent abundance. All statistical analyses in panels e to h and m were performed by one-way ANOVA (Bonferroni test), with different letters denoting statistical significance between samples, per phospholipid species.

We defined the fatty acid compositions of various mouse niches and their changes following intranasal challenge with the Gram-negative human pathogen Acinetobacter baumannii (strain AB5075_UW) (see Text S1 in the supplemental material). Overall, the fatty acid abundance in mouse plasma decreased following infection, with the most dramatic changes being seen in 16:0, 18:1, and 18:2 species (Fig. 1a). In contrast, 20:4 was not affected by A. baumannii infection, potentially due to a role in immune activation, findings consistent with those in mice infected with Streptococcus pneumoniae (9). The liver is the primary site of host fatty acid synthesis, and its profiling revealed the most pronounced increases in 18:1 and 18:2 (Fig. 1b), possibly to replenish their deficit observed in plasma. The bronchoalveolar lavage (BAL) fluid represents the primary bacterial challenge site, where 16:0 is the predominant fatty acid (Fig. 1c). The 16:0 abundance decreased marginally following infection, whereas that of 18:1 increased. Finally, we assayed the pleural lavage (PL) fluid, a sterile niche in uninfected animals, with fatty acid levels below the limit of detection (Fig. 1d). A. baumannii colonization resulted in the subsequent identification of 16:0, 18:2, 18:0, 18:1, and 20:4 species (in order of abundance). Similar to that observed in mouse plasma, the increase in 20:4 in the PL fluid may be due to a contribution to immune modulation. Overall, these findings indicate that A. baumannii necessitates adaptation to vastly different lipid landscapes when colonizing distinct host niches. The changes in the fatty acid profiles in these niches following infection may be a result of the host response to infection, in combination with the bacterium acquiring and secreting fatty acids during proliferation.

10.1128/mBio.00928-21.1 TEXT S1 Supplementary methods. Download Text S1, DOCX file, 0.03 MB. Copyright © 2021 Adams et al. 2021 Adams et al. https://creativecommons.org/licenses/by/4.0/ This content is distributed under the terms of the Creative Commons Attribution 4.0 International license.

To ascertain the potential exchange of fatty acids between A. baumannii and the host environment, we analyzed the lipidome of A. baumannii isolated from the pleural cavity and compared it to that of A. baumannii cultured in Luria-Bertani (LB) medium or M9 minimal medium (Text S1). Unlike the BAL fluid, the PL fluid is sterile in uninfected animals, which eliminates the contamination of the sample by non-A. baumannii bacterial species. Furthermore, as the level of A. baumannii colonization in this niche is high (>10^9^ CFU per sample), there is no need for tissue disruption or the removal of erythrocytes, and host immune cells can easily be depleted from the lavage fluid via centrifugation (Text S1). We studied the most abundant A. baumannii phospholipids, phosphatidylethanolamine (PE) and phosphatidylglycerol (PG), by liquid chromatography-mass spectrometry (LC-MS) (Fig. 1e and f). The analysis of A. baumannii cultured in M9 medium revealed that the bacterium does not readily synthesize phospholipids with acyl chains that harbor multiple double bonds (Fig. 1g and h). Examples of these phospholipid species with polyunsaturated acyl chains, which were most likely host acquired, include PE 36:3 (18:1 plus 18:2; 5.6 × 10^3^-fold increase [PL fluid as compared to M9 medium]), PE 36:4 (16:0 plus 20:4; 1.8 × 10^4^-fold increase [PL fluid as compared to M9 medium]), PG 34:3 (16:0 plus 18:2; 1.5 × 10^3^-fold increase [PL fluid as compared to M9 medium]), and PG 36:3 (18:1 plus 18:2; 9.1 × 10^2^-fold [PL fluid as compared to M9 medium]) (Fig. 1g and h). These data are consistent with the relative abundances of 18:2 and 20:4 in the PL fluid (Fig. 1d). Furthermore, we previously reported that 20:4 is preferentially incorporated into the PE pool of A. baumannii, with 16:0 occupying the other position on the phospholipid, thereby generating PE 36:4 (10). Similarly, A. baumannii cultured in the presence of 18:3 combines this exogenous fatty acid with 16:0 to generate PE 34:3 (11). To illustrate that the bacterial isolation procedure eliminated eukaryotic contamination, we compared the LC-MS signal intensities of LB medium-cultured cells and the PL fluid samples of infected and uninfected mice (Fig. 1i to l). In particular, we interrogated the species that were present in a high abundance in A. baumannii from PL fluid samples (Fig. 1i and j) and those of similar abundances between *in vitro* and *in vivo* A. baumannii cells (Fig. 1k and l). Noteworthy, PG 34:3 (16:1 plus 18:2) and PG 36:3 are unusual species not commonly found in mammalian cells (LipidMAPS database) (12, 13) or in A. baumannii grown in LB or M9 medium (Fig. 1j) (10). Hence, its presence in A. baumannii isolated from the mouse pleural cavity is a strong indicator of A. baumannii 18:2 acquisition from the host, reminiscent of 18:1 acquisition by S. aureus in mouse thigh tissue (14). The enhanced incorporation of 18:2 fatty acids in the A. baumannii PE and PG pools affected the total number of unsaturated fatty acids in A. baumannii from the PL fluid, by 1.4- and 1.3-fold (PE) and 1.04- and 1.06-fold (PG) compared to those cultured in M9 and LB media, respectively (Fig. 1m).

**FIG 2:**
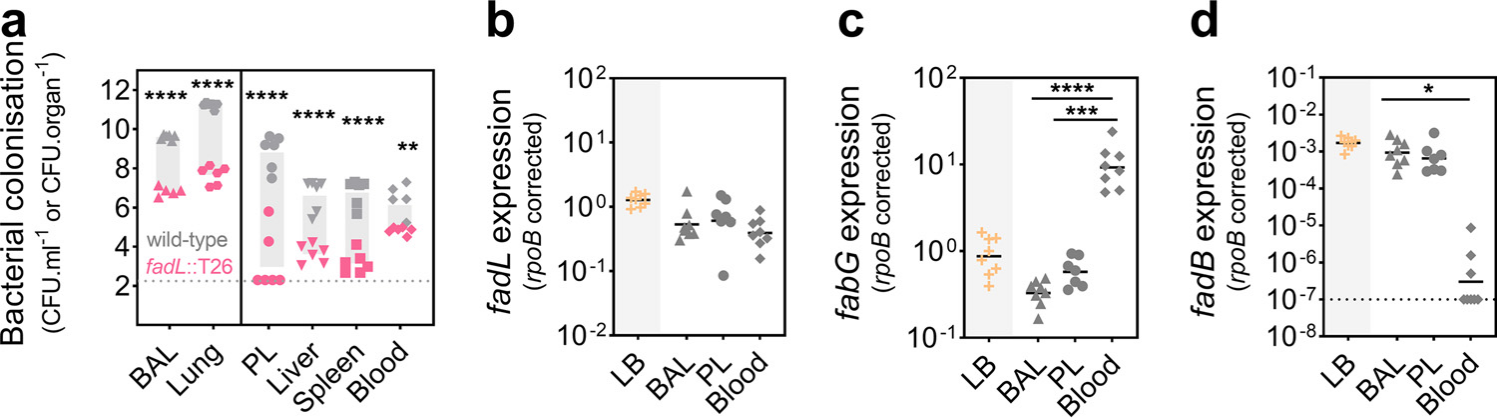
The niche-specific balance between fatty acid acquisition and *de novo* synthesis. (a) The role of FadL-mediated fatty acid acquisition in A. baumannii colonization was examined following intranasal challenge of 9-week-old female BALB/c mice with 3 × 10^8^ CFU of A. baumannii AB5075_UW (wild type) and the *fadL* mutant (*fadL*::T26). The differences between the two groups (geometric means) are indicated with gray bars. Statistical analyses were performed using one-way ANOVA (Bonferroni test). **, *P* ≤ 0.01; ****, *P* ≤ 0.0001. (b to d) The gene expression levels of *fadL* (b), *fabG* (c), and *fadB* (d), corrected against those defined for *rpoB*, in A. baumannii AB5075_UW cultured in Luria-Bertani (LB) medium or isolated from the bronchoalveolar lavage (BAL) fluid, pleural lavage (PL) fluid, or blood were examined by quantitative reverse transcription-PCR (qRT-PCR). The dotted lines indicate the limits of detection (a and d). All statistical analyses were performed using one-way ANOVA (Bonferroni test). *, *P* ≤ 0.05; ***, *P* ≤ 0.001; ****, *P* ≤ 0.0001.

To study the relative contribution of fatty acid acquisition to A. baumannii virulence, we challenged mice intranasally with wild-type AB5075_UW and a *fadL* mutant derivative (A. baumannii *fadL*::T26) and compared their invasion in diverse niches. The *fadL* mutant was significantly impaired in the colonization of the bronchoalveolar lumen, lung tissue, pleural cavity, liver, and spleen, ranging from 6.5 × 10^2^-fold (BAL fluid) to 2.0 × 10^4^-fold (PL fluid) (Fig. 2a). In contrast, colonization of the *fadL* mutant was only marginally lower from that of the wild type in the blood (1.6 × 10^1^-fold). Although these phenotypes are most likely related to the role in fatty acid acquisition, FadL is a surface-exposed protein that has also been shown to play a role in adherence to host cells in Haemophilus influenzae (15). Hence, from these data alone, we cannot rule out a multifactorial cause behind the observed colonization differences.

To delineate the role of exogenous fatty acid acquisition relative to *de novo* synthesis, we examined the transcription levels of *fadL* and *fabG* (a critical FASII member) in A. baumannii from the BAL fluid, PL fluid, and blood (Text S1 and Table S1). Overall, *fadL* transcription levels were similar in distinct host niches (Fig. 2b). Although the *fabG* expression level was significantly higher in A. baumannii from the PL fluid than from the BAL fluid (1.8-fold), the *fabG* transcription level was 17-fold higher in A. baumannii from the blood than from the PL fluid (Fig. 2c). In combination with FadL being nearly superfluous during A. baumannii bacteremia, this is a strong indicator that A. baumannii relies heavily upon *de novo* fatty acid synthesis while residing in the blood. In other niches, A. baumannii is likely to augment the intracellular pool of fatty acids with exogenously acquired fatty acids through uptake via FadL. To gain greater insights into the intracellular A. baumannii lipid status, we also assayed the expression of *fadB* (a critical component of the β-oxidation pathway), as its expression in A. baumannii is associated with an intracellular fatty acid surplus (10). Interestingly, whereas *fadB* expression levels in A. baumannii were comparable in the PL and BAL fluids, in the blood, expression decreased by approximately 700-fold (Fig. 2d). This is consistent with A. baumannii experiencing fatty acid limitation and relying upon synthesizing its own fatty acids in this niche. Transcriptional profiling of *fadL*, *fabG*, and *fadB* in A. baumannii cultured in LB medium indicated that the bacterium balances *de novo* synthesis and fatty acid uptake/recycling when grown in this medium, as it was analogous to that observed in A. baumannii in the PL fluid (Fig. 2b to d). Noteworthy, FadL has been shown to play roles other than in the acquisition of exogenous fatty acids to supplement the bacterial membrane. For example, in Salmonella enterica, FadL-mediated fatty acid acquisition influences the expression of the pathogenicity island 1 type III secretion system (16). Furthermore, FadL has been shown to exert dual roles in Haemophilus influenzae, these being fatty acid acquisition and host cell adherence (15).

10.1128/mBio.00928-21.2 TABLE S1 Oligonucleotides used in the study. Download Table S1, DOCX file, 0.02 MB. Copyright © 2021 Adams et al. 2021 Adams et al. https://creativecommons.org/licenses/by/4.0/ This content is distributed under the terms of the Creative Commons Attribution 4.0 International license.

### Conclusions.

A. baumannii is an opportunistic pathogen with limited host adaptation traits. Here, we have illustrated that A. baumannii utilizes environmental fatty acids to promote colonization in various niches of the host, which include nonself fatty acids, such as 18:2, to generate a unique phospholipid profile. Host fatty acid profiling revealed a decrease in most fatty acid species in the plasma following infection with A. baumannii. This could be a host-mediated response to infection or depletion due to A. baumannii acquisition during the early stages of bacteremia. However, the relative competitiveness of the *fadL* mutant compared to the wild type in the blood suggests the former. Despite the relatively high abundance of fatty acids in the plasma, these do not appear to be bioavailable to A. baumannii, potentially as a result of sequestration by plasma lipid binding proteins such as albumin and low/high-density lipoproteins. Fatty acid synthesis is an attractive target for the development of new antimicrobial strategies, and our work has underscored the critical need to understand microbial lipid homeostasis before this can be deemed suitable. Overall, FASII-targeting antibiotics are likely to be most effective against A. baumannii bloodstream infection.
