# Supplementary material for: To Make or Take: Bacterial Lipid Homeostasis during Infection
Source: mBio. 2021 Jun 17;12(3):e00928-21. doi: 10.1128/mBio.00928-21 (PMC8262940; doi:10.1128/mBio.00928-21)
Supplement: TEXT S1 [file mbio.00928-21-s0001.docx]

## Supplementary Methods

**To make or take: bacterial lipid homeostasis during infection**

Felise G. Adams^1^, Claudia Trappetti^2^, Jack K. Waters^1^, Maoge Zang^1^, Erin B. Brazel^2^, James C. Paton^2^, Marten F. Snel^3^ and Bart A. Eijkelkamp^1*^

**Bacterial strains, chemicals, media and growth**

The strains included in the study are *Acinetobacter baumannii* AB5075_UW and its *fadL* mutant derivative, purchased from the Manoil Laboratory (1) and confirmed by PCR. All chemicals were purchased from Sigma-Aldrich unless otherwise indicated. *A. baumannii* strains were grown in Luria Bertani (LB; 1% tryptone (BD Bacto), 0.5% yeast extract (BD Bacto) and 0.5% sodium chloride) or M9 (0.4% succinate) media and grown to an optical density at 600 nm of 0.7 for subsequent transcriptional profiling or lipid analyses.

**Animal experiments**

Nine-week old outbred female BALB/c mice were anaesthetized by intraperitoneal injection of ketamine (100 μg.g body weight^-1^) and xylazine (10 μg.g body weight^-1^), followed by intranasal administration of 40 μl bacterial suspension containing approximately 3 × 10^8^ CFU as described previously (2, 3). The challenge dose was confirmed retrospectively by serial dilution and plating for enumeration. At 24 hours post-challenge, mice were humanely euthanized by CO_2_ asphyxiation. The blood was collected (500 µL) via a cheek bleed and the pleural cavity was lavaged through injecting 1 mL sterile phosphate buffed saline (PBS) through the diaphragm. The lungs were lavaged through the trachea with 1 mL sterile PBS. Pulmonary vasculature was perfused by infusion of sterile PBS through the heart, and lungs, liver and spleen were subsequently excised. Tissues (complete organ) were homogenized in 1 mL PBS (Precellys Homogeniser) and a small fraction (20 µL) of the samples were serially diluted and plated for bacterial counts.

For the isolation of *A. baumannii* from the BAL (qRT-PCR), PL (qRT-PCR and lipid analyses) or blood (qRT-PCR), samples were diluted 10-fold into ice-cold PBS and subjected to centrifugation at 300 × *g* for 10 minutes at 4℃. This step allows for the pelleting of eukaryotic cells and tissue debris, whilst leaving the majority of the bacterial cells in the supernatant (4). Only 9 mL of supernatant was transferred to a new 15 mL tube, thereby preventing the inclusion of eukaryotic cells in the subsequent analyses. The bacteria in the supernatant were then pelleted by centrifugation at 3,000 × *g* for 15 minutes, washed 2 times in PBS (centrifugation at 3,000 × *g* for 15 minutes) and transferred to QiaZol (Qiagen) for transcriptional analyses. Alternatively, pellets from 3 mice were pooled and subsequently processed for lipid extraction. All samples, including those of uninfected mice, were processed using the same volumes, irrespective of bacterial colonisation levels.

All procedures performed in this study were conducted with a view to minimizing the discomfort of the animals. All experiments were approved by the University of Adelaide Animal Ethics Committee (Animal Welfare Assurance number A5491-01; project approval number S-2019-080) and were performed in strict adherence to guidelines dictated by the Australian Code of Practice for the Care and Use of Animals for Scientific Purposes.

## Lipid analyses

To extract mouse lipids from liquid samples (*i.e.* plasma, BAL and PL), samples were first subjected to low speed centrifugation (300 × *g* for 10 minutes at 4℃) to remove eukaryotic cells. The supernatants were transferred to a new vessel and subjected to high speed centrifugation (14,000 × *g* for 5 minutes) to eliminate bacterial contamination. The supernatants, free of eukaryotic and bacterial cells were then analysed by GC-MS. Since the liver is processed as a solid tissue for lipid analyses, bacterial elimination was not pursued. All samples were processed by addition of 1 ml of chloroform:methanol (2:1; v/v), vigorously mixing for 2 minutes and subsequently incubated at RT for 10 minutes. Following the addition of 200 μl 1.5% NaCl, the suspension was mixed vigorously for 1 minute and centrifuged at 6,000 × *g* for phase-separation. The lower phase was recovered and concentrated via nitrogen evaporation. All samples were stored at -20^o^C prior to gas chromatography - mass spectrometry (GC-MS) analysis. To generate fatty acid methyl esters (FAMEs), concentrated lipid samples were resuspended in 1:1 chloroform and trimethylsulfonium hydroxide (TMSOH) and subsequently analysed using an Agilent 7890A GC system with a 30 m Agilent DB-FastFAME column (Agilent Technologies). Mass spectrometry was completed using a coupled Agilent 5975C MSD system (Agilent Technologies). FAME species were differentiated and determined by comparing to the FAME mix c4-24 standard (Sigma Aldrich). Data analysis was completed using the Agilent MassHunter Qualitative Navigator software (Agilent Technologies).

Bacterial cell pellets from 20 mL LB or M9 cultures (~2 × 10^9^ CFU per sample), or 2.4 mL of mouse PL fluid (800 μL pooled from 3 mice), were resuspended in 50 μl of 1.5% NaCl buffer, followed by the addition of 1 ml of chloroform:methanol (2:1; v/v), vigorous mixing for 2 minutes and incubation at RT for 10 minutes. Following the addition of 200 μl 1.5% NaCl, the suspension was mixed vigorously for 1 minute and centrifuged at 6,000 × *g* for phase-separation. The lower phase was recovered and concentrated via nitrogen evaporation. All samples were stored at -20^o^C prior to liquid chromatography - mass spectrometry (LC-MS) analysis. Lipidomics analysis was performed using a LC-MS system comprising of an Acquity I-class UPLC system (Waters Corporation, Milford, MA, USA) fitted with 100 mm length, 2.1 mm inner diameter HSS T3 analytical column (Waters Corporation, Milford, MA, USA) coupled to a Xevo G2-XS Q-Tof mass spectrometer (Waters Corporation, Wilmslow, UK). Lyophilised samples were all reconstituted in 90 µL of 1:1 v/v acetonitrile (LC-MS grade):propan-2-ol (LC-MS grade). An injection volume of 5 µL was used for both positive and negative ion mode acquisitions. Mobile phase A was made up by combining 800 mL of water (LC-MS grade) with 1.26 g of ammonium formate (LC-MS grade, Sigma, Castlehill, NSW, Australia) and 1200 mL of acetonitrile.  Mobile phase B was a mixture of 200 mL acetonitrile and 1800 mL of propan-2-ol, to this was added 1.26 g of ammonium formate dissolved in 2 mL of water.  The flowrate used was 0.4 mL/min. The solvent gradient started at 40% B rising to 43% over 2 minutes before being stepped up to 50%. From 50% the concentration of B was increased to 54% over 10 minutes at which point the concentration was stepped up to 70%. Finally, the percentage of B was raised to 99% over 6 minutes before being returned to starting conditions of 40% and left to re-equilibrate for 2 minutes.

Mass analysis was performed in MS^E^-mode over an *m/z* range of 100 – 1200, in this mode the collision energy alternates every 100 ms between 6 V and a 20 V – 30 V ramp.  At the lower energy intact lipid ions are measured whereas at elevated energy diagnostic fragment ions are generated to aid lipid identification.

All data analysis was performed using SkyLine software (v20.2) for relative quantification (5). Lipids were identified based on exact precursor mass, diagnostic fragment ions and chromatographic retention time.

**qRT-PCR**

For RNA extraction and qRT-PCR analysis of *A. baumannii* bacteria, cells were lysed in QiaZol (Qiagen) as described previously (6). Following the addition of chloroform and phase separation, RNA was extracted and purified using a RNeasy Mini Kit (Qiagen), with on-column DNase I treatment, according to the manufacturer's instructions. qRT-PCR was performed using the SuperScript III One-Step RT-PCR kit (Thermo Fisher Scientific) on a QuantStudio 7 Flex System (Thermo Fisher Scientific). Transcription levels of genes were corrected to those obtained for the *rpoB* housekeeping gene. Oligonucleotide sequences are listed in (**Table S1**).

**References**

1. Gallagher LA, Ramage E, Weiss EJ, Radey M, Hayden HS, Held KG, Huse HK, Zurawski DV, Brittnacher MJ, Manoil C. 2015. Resources for genetic and genomic analysis of emerging pathogen *Acinetobacter baumannii*. J Bacteriol 197:2027-35.

2. Alquethamy SF, Adams FG, Naidu V, Khorvash M, Pederick VG, Zang M, Paton JC, Paulsen IT, Hassan KA, Cain AK, McDevitt CA, Eijkelkamp BA. 2020. The role of zinc efflux during *Acinetobacter baumannii* infection. ACS Infect Dis 6:150-158.

3. Alquethamy SF, Khorvash M, Pederick VG, Whittall JJ, Paton JC, Paulsen IT, Hassan KA, McDevitt CA, Eijkelkamp BA. 2019. The role of the CopA copper efflux system in *Acinetobacter baumannii* virulence. Int J Mol Sci 20.

4. Eijkelkamp BA, Morey JR, Neville SL, Tan A, Pederick VG, Cole N, Singh PP, Ong CY, Gonzalez de Vega R, Clases D, Cunningham BA, Hughes CE, Comerford I, Brazel EB, Whittall JJ, Plumptre CD, McColl SR, Paton JC, McEwan AG, Doble PA, McDevitt CA. 2019. Dietary zinc and the control of *Streptococcus pneumoniae* infection. PLoS Pathog 15:e1007957.

5. Adams KJ, Pratt B, Bose N, Dubois LG, St John-Williams L, Perrott KM, Ky K, Kapahi P, Sharma V, MacCoss MJ, Moseley MA, Colton CA, MacLean BX, Schilling B, Thompson JW, Alzheimer's Disease Metabolomics C. 2020. Skyline for small molecules: A unifying software package for quantitative metabolomics. J Proteome Res 19:1447-1458.

6. Hassan KA, Jackson SM, Penesyan A, Patching SG, Tetu SG, Eijkelkamp BA, Brown MH, Henderson PJ, Paulsen IT. 2013. Transcriptomic and biochemical analyses identify a family of chlorhexidine efflux proteins. Proc Natl Acad Sci U S A 110:20254-9.
