# Supplementary material for: To Make or Take: Bacterial Lipid Homeostasis during Infection
Source: mBio. 2021 Jun 17;12(3):e00928-21. doi: 10.1128/mBio.00928-21 (PMC8262940; doi:10.1128/mBio.00928-21)
Supplement: TABLE S1 [file mbio.00928-21-st001.docx]

**Table S1: Oligonucleotides used in the study**

| **Primer name** | **Sequence (5' – 3')** | **Reference/source** |
| --- | --- | --- |
| q5075_3108_F | AACAAGGTGCAGGTTTGGCT | (1) |
| q5075_3108_R | ATCCCAATCATCTTCCGACA | (1) |
| q5075_3572_F | TCGCGGCTGTAAAAGCTAAT | (1) |
| q5075_3572_R | CTATTGAAGATTTCGTGAAC | (1) |
| qABUW_0724_F | CAATCGCAGTGGCTGATAAA | This study |
| qABUW_0724_R | ATGGCGAATGACTACTACTT | This study |
| *rpoB*_F | CTCACCGCCGACTTTGTTAT | This study |
| *rpoB*_R | ACCCGCTGGGTGTACCATCT | This study |
| ABUW_mut_0724_F | AGAAGCAATCCGTTCTGT | This study |
| ABUW_mut_0724_R | TACATGGAGTTATCAATG | This study |

1. Jiang JH, Hassan KA, Begg SL, Rupasinghe TWT, Naidu V, Pederick VG, Khorvash M, Whittall JJ, Paton JC, Paulsen IT, McDevitt CA, Peleg AY, Eijkelkamp BA. 2019. Identification of novel *Acinetobacter baumannii* host fatty acid stress adaptation strategies. MBio 10:1 e02056-18.
